# Addition of Popular Exogenous Antioxidant Agent, PBN, to Culture Media May Be an Important Step to Optimization of Myogenic Stem/Progenitor Cell Preparation Protocol

**DOI:** 10.3390/antiox10060959

**Published:** 2021-06-15

**Authors:** Magdalena Nowaczyk, Agnieszka Malcher, Agnieszka Zimna, Wojciech Łabędź, Łukasz Kubaszewski, Wojciech Barczak, Błażej Rubiś, Natalia Rozwadowska, Maciej Kurpisz

**Affiliations:** 1 Institute of Human Genetics, Polish Academy of Sciences, 60-479 Poznan, Poland; magdalena.przybyl@igcz.poznan.pl (M.N.); agnieszka.malcher@igcz.poznan.pl (A.M.); agnieszka.zimna@igcz.poznan.pl (A.Z.); natalia.rozwadowska@igcz.poznan.pl (N.R.); 2 Department of Orthopedics and Traumatology, W. Dega University Hospital, Poznan University of Medical Sciences, 61-545 Poznan, Poland; wlabedz23@gmail.com (W.Ł.); pismiennictwo1@gmail.com (Ł.K.); 3 Radiobiology Lab, The Greater Poland Cancer Centre, 61-866 Poznan, Poland; wojciech.barczak@oncology.ox.ac.uk; 4 Department of Clinical Chemistry and Molecular Diagnostics, Poznan University of Medical Sciences, 61-701 Poznan, Poland; blazejr@ump.edu.pl

**Keywords:** α-phenyl-N-tert-butyl nitrone, heart failure, skeletal muscle derived stem cells, apoptosis, oxidative stress, post-infarction heart

## Abstract

The aim of the study was to modify human skeletal muscle-derived stem/progenitor cells (SkMDS/PCs) and demonstrate the optimal cell preparation protocol for application in post-infarction hearts. We used conditioned SkMDS/PC culture medium with α-phenyl-N-tert-butyl nitrone (PBN). SkMDS/PCs were cultured under hypoxic conditions and the results were compared to the standard ones. We observed a significant increase of CD-56 positive phenotypic marker the ability to form functional myotubes, increase in the proportion of young cells in cell primary suspensions, and a decrease in the percentage of apoptotic cells among PBN-conditioned cells in normoxia an hypoxia. We also observed significantly higher levels of *SOD3* expression; maintained expression of *SOD1*, *SOD2*, and *CAT*; a higher level of *BCL2* gene expression; and a rather significant decrease in *Hsp70* gene expression in PBN-conditioned SkMDS/PCs compared to the WT population under hypoxic conditions. In addition, significant increase of myogenic genes expression was observed after PBN addition to culture medium, compared to WT population under hypoxia. Interestingly, PBN addition significantly increased the lengths of telomeres under hypoxia. Based on the data obtained, we can postulate that PBN conditioning of human SkMDS/PCs could be a promising step in improving myogenic cell preparation protocol for pro-regenerative treatment of post-infarction hearts.

## 1. Introduction

Human myogenic cells cultured in vitro present an attractive perspective for their application in regenerative medicine due to their unique self-propagation properties. In recent years, different stem/progenitor cell candidates have been explored to treat various tissue defects. This attracted worldwide attention towards new treatment approaches in cases of severe heart failure, a frequently occurrence in modern societies [1]. Direct injections of either autologous skeletal myoblasts or bone marrow-derived cells have been examined in clinical studies as an alternative cell source by which to replace cardiac muscle cells damaged due to ischemic episodes [2]. However, a direct administration of cell suspensions has been shown to generally be less effective and more difficult to control in terms of retention in the target site, or proper positioning after implantation, with weakened functioning in vivo under pathological conditions [3]. A lot of myogenic stem cell modification methods have been developed to help their adaptation in recipient tissues: selected gene over-expressions, micro RNA insertion and/or its inhibition, as well as in vitro culture modifications [4]. The aim of all these modifications is to improve their relatively low engraftment efficacy and tissue replacement capacity via cell pre-conditioning, extension of lifespan, and functioning in situ, as well as increasing their long-term beneficial effects.

We observed the problem of oxidative stress and a shift in redox balance between pro-oxidants and antioxidants, influencing the cell microenvironment. Studies on the generation of reactive oxygen species (ROS) in highly reactive molecules that can damage almost all cell structures critical for cell viability, activation, proliferation, and function within specialized tissues of a particular organ are of the utmost importance (carbohydrates, lipids, nucleic acids, and proteins) [5]. Additionally, it has been underscored that oxidative stress may represent a ground to change atrial pacing and postoperative atrial fibrillation. An increasing body of evidence has indicated that the excessive production of reactive oxygen species released after heart infarction, or due to application of extracorporeal circulation, could be involved in structural and functional myocardial impairments due to perfusion-reperfusion episodes [6].

Nitrone-based spin trapping compounds have been used in experimental animal models to protect pathologies associated with accelerated aging, endotoxaemia, ischaemia/reperfusion injury, certain xenobiotics, and physical trauma. Furthermore, nitrones affect pathophysiological mediators in the peripheral organ systems and central nervous system. It has been shown that nitrones affect cellular redox status and oxidatively sensitive enzymatic systems, but the precise mode of nitrone action has not been yet elucidated [7].

One of the two studies to date on the effects of α-phenyl-N-tert-butyl nitrone (PBN) on myoblasts showed that an ROS trapping agent combined with PBN led to the reduction of redox potentials and enhanced differentiation in an immortalized mouse myoblast cell line (C2C12). Under most oxidative conditions, quantitative PCR has shown a significant decrease in myogenic basic helix-loop-helix transcription factor expression compared to cultures treated with phenyl PBN or grown in 6% O_2_. Thus, it has been claimed that oxidative intracellular environments impair myoblast differentiation, while reducing environments favorable for myogenesis [8]. The few groups that have used PBN in cardiovascular entities have shown that treatment with a PBN analog, N-tert-Butyl-α-(2-sulfophenyl) nitrone (2-SPBN) produced a sublethal endotoxin shock in horses that was reported to be effective in normalizing heart and respiratory rates [9]. PBN has also been shown to possess vasodilatating ability. In a perfused rat heart model, PBN showed coronary vasodilation when the perfusate contained PBN higher than 3 mM [10]. Spin-trapping experiments using PBN as a spin trap to detect free radical formation in a dog coronary artery occlusion/reperfusion model were successful in demonstrating the myocardial release of free radicals immediately after reperfusion. The investigators also noticed that the recovery of contractile function (measured as systolic wall thickening) after reperfusion was significantly greater in dogs given PBN than in controls [11].

Based on these limited data we decided first to study PBN pre-conditioning in human SkMDS/PC primary suspensions cultured in vitro, in order to create a new myogenic stem cells preparation protocol for prospective use in regenerative approaches to post-infarction hearts. Thus, we wanted to reduce the cellular stresses that occur during cell preparation and in in vitro culture by supplementing a cytoprotective agent (PBN). At the same time, thanks to the properties of the spin trap, we determined to induce cellular protection by stimulating anti-apoptotic, anti-aging, proliferative and genetic pro-regenerative modalities in post-infarction hearts.

## 2. Materials and Methods

### 2.1. Human Skeletal Muscle-Derived Stem/Progenitor Cells (SkMDS/PCs) Isolation

SkMDS/PCs were isolated from tissue fragments derived after surgical intervention in abdominal rectus muscle area. For this purpose, the study was approved by the Ethical Local Committee (Medical University of Poznan, Poland, permission no. 818/13) and was conducted in accordance with the principles of Good Clinical Practice, with written consent obtained from the patients involved. All methods used in this study involving human biological material adhered to the principles outlined in the Declaration of Helsinki.

Isolation of SkMDS/PCs was conducted according to a previously modified technique [4,12]. Briefly, pre-purified and fragmented tissue was enzymatically digested with collagenase type II (Sigma-Aldrich, Saint Louis, MO, USA) for 45 min at 34 °C, then filtered through mesh with a 70 µm diameter, neutralized with Hanks’ balanced salt solution, and centrifuged for 10 min with 1200 rpm at room temperature. The cells were then cultured in standard Dulbecco’s modified Eagle’s medium with 4.5 g/L glucose. Cell were supplemented with 20% fetal bovine serum (Lonza Group, Base, Switzerland), 1% antibiotics (Lonza Group, Basel, Switzerland), 1% ultraglutamine (Lonza Group, Basel, Switzerland), and a basic fibroblast grow factor (bFGF) (Sigma-Aldrich, Saint Louis, MO, USA). Tissue culture flasks were coated with gelatine. The cells were incubated in standard (95% humidity in 5% CO_2_ and 21% O_2_ at 37 °C) or under hypoxic in vitro conditions (95% humidity, 5% CO_2_ and 3% O_2_, at 37 °C). After every 2–3 days of culture, the cell confluence was observed, and when required the digested cell suspensions were transferred to another culture flask coated with gelatine.

The medium was changed every other day, and cells were passaged using 0.25% trypsin with PBS (Lonza Group, Basel, Switzerland). After the 4th passage, when culturing potential of SkMDS/PCs was optimal, the medium was supplemented with 80 µM PBN (in 100 µL of DMSO), and at 24 h (after first conditioning) cells were placed under hypoxia (3% of oxygen). The experimental procedures were performed after the 7th cell passage, after about 1 week of in vitro culturing and a minimum of 7 days after the beginning of culturing under hypoxic conditions. Every other day the medium was changed when the cell passage was performed, and the appropriate amount of PBN was then added. Passages were performed when the cell confluence reached approximately 75–90% of monolayer surface, which was microscopically assessed. The experiments for both in vitro culture conditions were performed at the same time because the culture proliferation rates were comparable and hypoxia did not slow down cell division or growth.

### 2.2. Hypoxia Optimization

The conditions for hypoxic in vitro cultures of the SkMDS/PCs were designed previously, by plotting the oxygen concentration curve to compare hypoxia levels in myogenic cells transplanted into post-infarcted hearts of severe combined immunodeficient (SCID) mice [12].

### 2.3. Evaluation of CD56 Positive Cells

Cytometry was used to determine the purity of the human SkMDS/PC population under study. The cell cultures were first evaluated with flow cytometry using anti-neural cell adhesion molecule (CD56) PC5 conjugate (Beckman Coulter, Brea, CA, USA). Briefly, 2.5 × 10^5^ cells were harvested, centrifuged, and resuspended in 100 μL PBS with 2% FBS and 10 μL of antibodies (1:200 dilution). After 20 min incubation, cells were centrifuged, resuspended in PBS with 2% FBS, and further analyzed (Beckman Coulter, Brea, CA, USA).

### 2.4. The Cell Differentiation Potential for Myotube Formation

To estimate the human skeletal muscle stem/progenitor cells differentiation potential, in vitro cultures were kept under the cell differentiation protocol regime. Cells were cultured in 6-well plates, and 1 mln cells were examined per studied variant. SkMDS/PCs were cultured in differentiating medium composed of 1% antibiotics (Lonza Group, Basel, Switzerland), 1% ultraglutamine (Lonza Group, Basel, Switzerland), and 2% horse serum (Sigma-Aldrich, Saint Louis, MO, USA) for 1 week. The fusion index (FI) of all the cell populations under study was determined. The differentiated cell populations were fixed in freezing methanol:acetic acid (3:1) solution and stained using a Giemsa solution (Merck, Darmstadt, Germany). Photographs were taken using a standard light microscope. All the cell nuclei showing microscopic images were counted (≥450 nuclei). FI was defined as the ratio of the number of nuclei present in the differentiated myotubes (Nd) to the total number of nuclei × 100. FI = Nd/(Nd + Nnd).

### 2.5. Cell Senescence Assay

Senescence-associated expression of β-galactosidase (SA-β-Gal) activity was evaluated using the cell senescence detection kit (BioVision, Milpitas, CA, USA). A kit was designed to chemically detect SA-β-Gal activity in in vitro cultured cells. SA-β-GL is present only in senescent cells and has not been found in pre-senescent, quiescent, or immortal cells. SkMDS/PCs were cultured in 6-well dishes, and washed once with PBS. Next, cells were fixed with Fixative Solution for 15 min at room temperature. After incubation, SkMDS/PCs were washed twice with PBS and stained with 50 µL of 20 mg/mL X-gal in 1 mL of Staining Solution and Staining Supplement. After overnight incubation at 37 °C, cells were washed with water and 1% acid alcohol, and stained with eosin for 5 min for better cell visualization. SkMDS/PC were observed and counted under a light microscope (Leica DMi8).

### 2.6. Detection of Apoptosis

Cell samples were collected and examined for the studied SkMDS/PCs populations (WT and PBN-conditioned in standard and hypoxic in vitro culture). Using an Annexin V-FITC Kit (Beckman Coulter, Fullerton, CA, USA) assay, we were able to detect the percentage of apoptotic cells in the SkMDS/PC populations under study. The cells were washed twice with cold PBS then resuspended in a binding buffer at a concentration of 5 × 10^6^ cells/mL. Then, a 100 μL cell sample was incubated with 1 μL Annexin-V-FITC solution and 5 μL propidium iodide (PI) for 15 min in the dark and analyzed via flow cytometry (Beckman Coulter, Brea, CA, USA). The main assumption of the test was the ability of Annexin V to bind inversely located phosphatidylserine. Annexin V is linked to fluorescein (FITC) and identifies apoptotic cells. Apoptotic cells were stained with Annexin V+/PI- for early apoptosis plus Annexin V+/PI+ for late apoptosis.

### 2.7. Gene Expression: Real-Time Polymerase Chain Reaction (RT-PCR)

SkMDS/PCs samples were harvested using trypsin and lysed in RNeasy Lysis Buffer (RLT) with beta-Mercaptoethanol. Total RNA was extracted using the AllPrep DNA/RNA/Protein Mini Kit (Qiagen, Hilden, Germany). A possible genomic DNA contamination was eliminated using a Turbo DNA-free kit (Invitrogen, Carlsbad, CA, USA). The purity and quality of isolated RNA was analyzed using a Nanodrop 2000 (Thermo Scientific, Waltham, MA, USA) and 1.5% agarose gel electrophoresis. Synthesis of complementary DNA (cDNA) was performed using 1.5 µg of total RNA via SuperScript IV Reverse Transcriptase (Invitrogen, Carlsbad, CA, USA). The resulting cDNA was diluted and validated through a PCR reaction with primers for β-actin gene The Real-Time PCR analyses were performed using iQ SYBR Green Supermix (Bio Rad, Warsaw, Poland). The single-reaction volume sample was 12.5 μL and contained: 2 μL of cDNA, 6.25 μL of 2 × iQ™ SYBR Green Supermix Reagent (BioRad, Hercules, CA, USA), and 1.25 μL of each 4 μM primer. The following conditions were used: 95 °C for 1 min, 45 cycles at 95 °C for 20 s, 60 °C for 20 s and 72 °C for 20 s. Genes: actin (*ACT*), glyceraldehyde 3-phosphate dehydrogenase (*GAPDH*), and hypoxanthine-guanine phosphoribosyltransferase (*HPRT*) were evaluated as reference genes, and all results were normalized quantitatively in each sample. The primers used have been displayed in Table 1. The expression of gene clusters was investigated determining superoxide dismutase (*SOD1*), superoxide dismutase 2, mitochondrial (*SOD2*), extracellular superoxide dismutase (*SOD3*), catalase (*CAT*), heat shock proteins *Hsp70*, forkhead Box O1 *FOXO1,* b-cell lymphoma 2 (*BCL2*) and sirtuin 1 (*SIRT1*). Next, early, myoblast determination protein 1 (*MYOD*) and late myogenesis genes myogenin (*MYOG*) were examined.

### 2.8. Assessment of Relative Telomere Length

Telomere length was assessed using two pairs of primers, one telomere-specific (*Telo*) and one a single copy gene-specific albumin *ALB* (Table 2), as previously described [13,14]. Briefly, specific primers with an efficiency close to 100% (98 ± 2%) were used in a SYBR Green based assay with FastStart Essential SYBR Green I Master (Roche Applied Science, Penzberg, Germany). Initial denaturation and polymerase activation (hot start) was performed at 95 °C for 10 min. The signal was detected over 45 cycles of 95 °C for 10 s, 61 °C for 10 s and 72 °C for 10 s. Melting analysis (65–95 °C; 0.2 °C resolution) was performed at the end of the reaction to verify specificity of the product. Telomere length was assessed using a qPCR system (LC 96; Roche Diagnostics, Basel, Switzerland) and calculated using the 2 ΔΔCq method.

### 2.9. Western Blot Procedure

For the Western blot experiments, cell pellets of SkMS/PCs were lysed in 8 M urea, 50 mM Tris–HCl, pH 8.0 with 1% SDS (1:1) containing protease inhibitor cocktail (Roche, Basel, Switzerland). The total protein concentration was determined using the Lowry method. A total of 50 μg of protein was separated on 4–20% Mini-PROTEAN TGX Stain-Free Protein Gels and electrotransferred under standard conditions (30 min) using Trans-Blot Turbo to a PVDF membrane (all from Bio-Rad, Hercules, CA, USA). The membrane was soaked with blocking buffer containing non-fat milk (Bio-Rad, Hercules, CA, USA). Immunodetection was performed using: the anti-forkhead box O1 (FOXO1A) antibody −70 kDa (ab52857); anti-heat shock protein70 (HSP70) antibody 1:1000, 70 kDa (ab31010); anti-myoblast determination protein 1 (MYOD) antibody 1:1000, −50 kDa; anti-oxidative stress defense (Catalase- CAT 60 kDa superoxide dismutase- SOD1 16 kDa, thioredoxin- TRX 12 kDa, smooth muscle actin- ACT 42 kDa) antibodies cocktail (ab179843) 1:200; and anti-hypoxanthine-guanine phosphoribosyltransferase (HPRT) antibody 1:1000, 25 kDa (ab109021) (Abcam, Cambridge, UK). The detection of the target protein was achieved by incubating the membrane with Clarity ECL, Western Blot substrate and analyzed with ChemiDoc XRS system (Bio-Rad, Hercules, CA, USA).

## 3. Results

### 3.1. Purity of Human Primary SkMDS/PCs Suspension Samples

**Figure 1:**
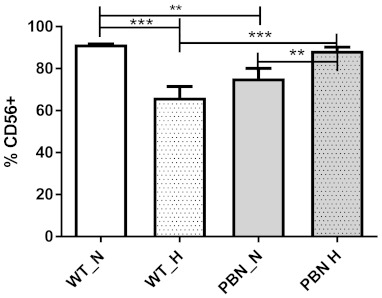
Percentage of CD56-positive skeletal muscle stem/progenitor cells (SkMDS/PCs) in in vitro culture. WT = wild-type SkMDS/PCs population, PBN = human SkMDS/PCs conditioned with N-tert-butyl-α-phenylnitrone; under standard in vitro culture conditions (N) and in hypoxia (H). ** significant at *p* value < 0.01, *** significant at *p* value < 0.001.

Prior to in vitro culturing with PBN antioxidant, human SkMDS/PCs samples were harvested then characterized by flow cytometry using an anti-CD56-specific antibody. The percentage of CD56-positive cells is shown in Figure 1.

The fluorescence-activated cell sorting (FACS) results indicated almost 90% CD56-positive cell populations when studied under the standard in vitro culture conditions, although under hypoxic conditions the expression level of the investigated CD56 marker was significantly lower. The cell confluence in both normoxia and hypoxia was comparable and the number of cells subjected to cytometry analysis was equal: 500,000 cells per sample. However, we observed that PBN conditioning led to a statistically significant increase (almost 10%) in CD56-positive cells under hypoxic conditions compared to the WT population (WT H) (*p* < 0.01). A statistically significant increase in purity of myogenic cell populations caused by PBN was not observed under standard in vitro culture conditions (hyperoxia).

### 3.2. Myotube Formation

**Figure 2:**
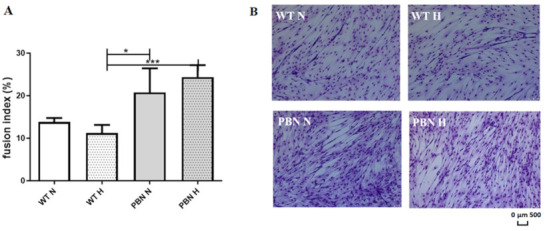
Percentages of fused nuclei in SkMDS/PCs-originated myotubes from the WT and PBN-treated in vitro cell culture shown as diagrams (**A**). SkMDS/PC fusion observed under a light microscope (**B**). Abbreviations: WT = wild-type non-treated SkMDS/PCs; PBN-SkMDS/PCs = cells cultured with antioxidant under N-standard and H-hypoxic in vitro culture conditions. * significant at *p* value < 0.05, *** significant at *p* value <0.001.

The population of PBN-treated SkMDS/PCs exhibited a superior ability for myotube formation, as shown in Figure 2. 

Culturing of SkMDS/PCs with PBN under standard in vitro culture conditions generated a 1.5-fold higher percentage of fused nuclei in this cell population compared to the WT non-treated cells; higher and statistically significant differences were observed in hypoxia where PBN conditioning led to improvement of myotube formation by more than 2-fold.

The fusion index, a ratio of the nuclei numbers in fused cells to the number of non-fused cells (×100) under standard and hypoxic conditions, was as follows: 13.84/11.25 in the WT control population, and 20.79%/24.42% in the respective PBN-conditioned myogenic cell population.

### 3.3. Senescence Test Based on SA-β-gal Activity

**Figure 3:**
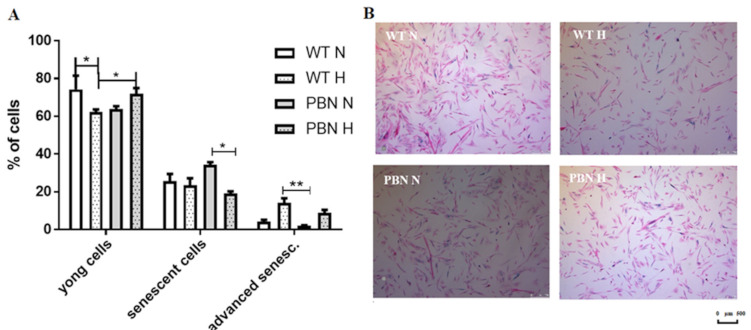
Percentages of young, senescent, and advanced senescent cells in the WT and PBN-treated SkMDS/PCs populations (**A**). X-gal senescence assay, SkMDS/PCs observed under a light microscope (**B**). WT = non-treated SkMDS/PCs, PBN-SkMDS/PCs cultured with antioxidant in standard (N) and hypoxic (H) culture conditions. Abbreviations: SkMDS/PCs = skeletal muscle-derived stem/progenitor cells; WT = wild-type non-treated SkMDS/PCs; PBN = SkMDS/PCs cultured in medium conditioned with tert-butyl-alpha-phenyl nitrone. * significant at *p* value < 0.05, ** significant at *p* value < 0.01.

The senescent SkMDS/PCs were positively stained for SA-β-gal (Figure 3). The percentages of young (SA-β-gal-negative) cells were different under the standard in vitro (74%) and hypoxic (62%) culture conditions (*p* < 0.05). In the case of PBN conditioning, the antioxidant contributed to a reverse such situation: 72% of young cells in hypoxia and 64% under standard in vitro culture conditions were observed. 

Higher percentages of the young myoblasts (SA-β-gal-negative cells) were observed in the PBN-treated cell population in hypoxia, as expected. We observed almost 10% more young cells in antioxidant-treated SkMDS/PCs compared to the WT control population (*p* < 0.05), and almost 1.5-fold less senescent and advanced senescent cells in this population. This indicates that PBN neutralizes the negative effects of in vitro culture under hypoxic conditions.

### 3.4. Apoptosis

**Figure 4:**
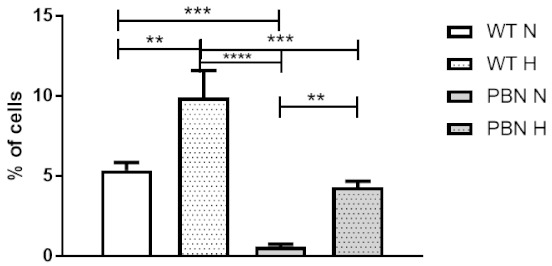
Cell apoptotic rate in the SkMDS/PCs populations under study: WT and PBN-conditioned cells under the standard (N) in vitro and hypoxic conditions (H). ** significant at *p* value < 0.01, *** significant at *p* value < 0.001, **** significant at *p* value < 0.0001.

We detected significantly lower cell mortality in the SkMDS/PCs cultured with PBN than in the WT respective control populations, as shown in Figure 4 (*p* < 0.001).

We observed a higher proportion of apoptotic cells under hypoxic conditions than in standard in vitro culture (*p* < 0.01), as was expected. However, using an antioxidant (PBN), we eliminated this negative effect of deficient oxygen concentration. PBN conditioning of SkMDS/PCs led to an almost 10-fold decrease in apoptosis level under standard in vitro culture conditions (*p* < 0.001) and a 2.5-fold decrease of apoptosis level under hypoxic culture conditions (*p* < 0.001) compared to the non-treated SkMDS/PCs populations.

### 3.5. Gene Expression

**Figure 5:**
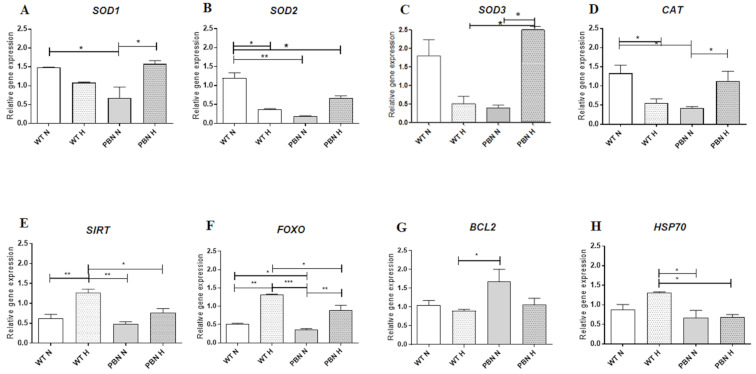
Expression levels of genes coding for superoxide dismutase 1 (*SOD1*) (**A**); manganese-dependent superoxide dismutase (*SOD2*) (**B**); extracellular superoxide dismutase (*SOD3*) (**C**); catalase (**D**); sirtuin1 (**E**); forkhead box O3 (**F**); B-cell lymphoma 2 (**G**), and heat shock proteins (**H**) in non-treated (WT) and PBN-conditioned (PBN) SkMDS/PC populations cultured in standard (N) and hypoxic (H) in vitro conditions. Abbreviations: SkMDS/PCs = skeletal muscle derived stem/progenitor cells; WT = wild-type non-treated SkMDS/PCs; PBN = SkMDS/PCs cultured in medium conditioned with tert-butyl-alpha-phenyl nitrone. * significant at *p* value < 0.05, ** significant at *p* value < 0.01, *** significant at *p* value < 0.001.

The expression of selected anti-apoptotic and anti-ageing genes was examined in human SkMDS/PC populations. The expression levels of genes coding for superoxide dismutases, catalase, sirtuin, *FOXO*, *BCL2*, and *Hsp70* are shown in Figure 5. 

We observed a decrease in expression level of superoxide dismutase 1 [Cu-Zn] (*SOD1*) caused by hypoxic conditions (1.3-fold) in the non-treated SkMDS/PCs population, as well as after addition of antioxidant (2.2-fold, *p* < 0.05) in the standard in vitro culture. This effect was neutralized by adding PBN under hypoxic conditions where expression of the *SOD1* gene was comparable with expression level in the WT SkMDS/PCs population (Figure 5A). 

The expression level of another dismutase gene, *SOD2*, was lower in hypoxic (almost 3-fold, *p* < 0.05) and standard in vitro PBN-conditioned (6-fold, *p* < 0.01) SkMDS/PCs as compared to the standard in vitro cultured cell populations. About a 1.7-fold decrease in this gene expression was demonstrated in PBN-treated SkMDS/PCs cultured under a hypoxic condition (*p* < 0.05) (Figure 5B) when compared to standard in vitro cultured myogenic cells.

In the case of *SOD3* gene expression (Figure 5C), we observed the highest expression of this gene in the PBN-treated cell population cultured under hypoxic conditions as compared to the non-treated cells cultured under the same conditions and to PBN-treated SkMDS/PCs under the standard in vitro culture (*p* < 0.05) (Figure 5C). As a result, we observed a higher expression of *CAT* gene in PBN-treated SkMDS/PCs under hypoxic conditions than in the population without such treatment (*p* < 0.05; Figure 5D).

In the next two panels of Figure 5E,F we confirm the comparable effect of in vitro culture on two anti-aging genes, *SIRT1* and *FOXO*. Expression of *SIRT1* (Figure 5E) and *FOXO* (Figure 5F) genes were highest in WT SkMDS/PCs in hypoxia not treated with PBN. Subsequently, an almost 2-fold lower level of expression was observed in corresponding PBN-treated SkMDS/PC populations (*p* < 0.05).

A positive effect on anti-apoptotic gene *BCL2* expression was demonstrated in the PBN-conditioned SkMDS/PC population under standard in vitro culture conditions, which was 1.5-fold higher than in the non-treated cell population (Figure 5G), while for anti-stress gene *Hsp70* the expression was almost 2-fold lower in the PBN-treated than for the WT SkMDS/PCs cultured under hypoxic conditions (*p* < 0.05) (Figure 5H).

**Figure 6:**
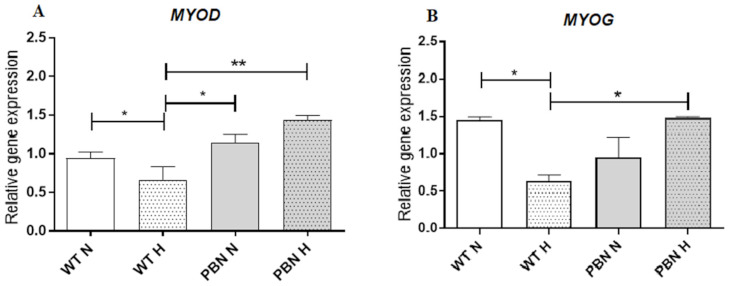
Expression levels of *MYOD* (**A**) and *MYOG* (**B**) genes in WT- non-treated myoblast populations and PBN-conditioned ones in standard in vitro (N) and hypoxic (H) in vitro culture conditions. * significant at *p* value < 0.05, ** significant at *p*-value < 0.01.

Conditioning of human SkMDS/PCs with antioxidant (PBN) when culturing under hypoxic conditions in vitro exerted positive effects on myogenic *MYOD* and *MYOG* gene expression. We observed a statistically significant 2-fold increase in expression level of the *MYOD* gene in hypoxic in vitro culture with PBN-conditioned medium when compared with the non-treated WT SkMDS/PC population in the same conditions (*p* < 0.001). It should be mentioned that exclusively used hypoxic conditions caused a significant decrease in *MYOD* expression (*p* < 0.05) (Figure 6A). We also observed a significant decrease in *MYOG* expression level (late myogenic transcription factor) after exposure to hypoxic conditions compared to the control cell culture under standard in vitro conditions (*p* < 0.05). Co-culturing with PBN led to an increase in *MYOG* expression, but this was not statistically significant when comparing to the control WT SkMDS/PCs population (Figure 6B). 

### 3.6. Relative Telomere Length

**Figure 7:**
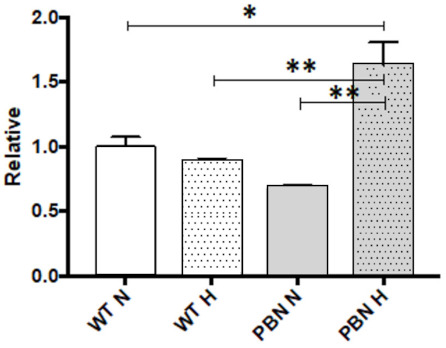
Relative length of SkMDS/PCs telomeres in PBN-treated cell populations: WT= wild-type SkMDS/PCs, PBN-SkMDS/PCs preconditioned with PBN under standard (N) and hypoxic (H) culture conditions. Abbreviations: SkMDS/PCs = skeletal muscle derived stem/progenitor cells; WT = wild-type non-treated SkMDS/PCs; PBN = SkMDS/PCs cultured in medium conditioned with tert-butyl-alpha-phenyl nitrone. * significant at *p* value < 0.05, ** significant at *p* value < 0.01.

The average relative telomere length in the PBN-treated SkMDS/PCs and their respective controls in different culture conditions was evaluated. Telomere length in the cells conditioned with PBN in hypoxia demonstrated a significant increase in comparison with the other cell populations under study. We observed a more than 1.5-fold increase in telomere length in the PBN-treated cells under hypoxic conditions vs. the WT non-treated control cell population (*p* < 0.05), a 1.7-fold increase compared to the WT SkMDS/PCs cultured under hypoxic conditions (*p* < 0.01) and more than a 2.3-fold greater length compared to the telomeres in the PBN-conditioned SkMDS/PCs in the standard in vitro culture (*p* < 0.01) (Figure 7).

### 3.7. Relative Protein Expression

**Figure 8:**
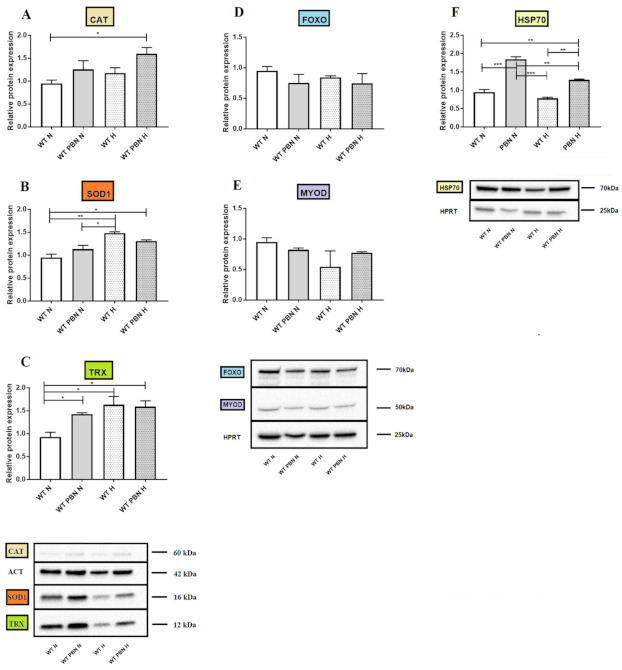
Amount of protein superoxide dismutase 1 (SOD1) (**A**, catalase (CAT) (**B**), thyredoxin (TRX) (**C**), forkhead box O3 (**D**), myoblast determination protein 1 (MYOD) (**E**), heat shock protein 70 (HSP70) (**F**), and housekeeping proteins: hypoxanthine-guanine phosphoribosyltransferase (HPRT) and actinin (ACT) in non-treated (WT) and PBN-conditioned (PBN) SkMDS/PCs populations cultured under standard (N) and hypoxic (H) in vitro conditions. Abbreviations: SkMDS/PCs = skeletal muscle derived stem/progenitor cells; WT = wild-type non-treated SkMDS/PCs; PBN = SkMDS/PCs cultured in medium conditioned with tert-butyl-alpha-phenyl nitrone. * significant at *p* value <0.05, ** significant at *p* value <0.01, *** significant at *p* value <0.001.

We also performed a Western blot analysis of some antioxidant (CAT, SOD1, THX) anti-aging (FOXO), myogenic (MYOD) and HSP70 protein products. We observed greater amounts of catalase, SOD1 and TRX in cell populations preconditioned with PBN cultured under hypoxic conditions compared to wild-type cells under standard in vitro conditions (*p* < 0.05). The use of hypoxic culture variants alone caused an increase in SOD1 (Figure 8B) and TRX (Figure 8C) protein (*p* < 0.05). PBN by itself increased the amount of TRX protein. The amount of HSP70 protein increased during PBN preconditioning (*p* < 0.001) (Figure 8F) as well as under hypoxic conditions (*p* < 0.01) (Figure 8). In general, it could be observed that the addition of PBN increased the amount of the respective protein product both under standard as well as hypoxic culture conditions.

## 4. Discussion

Spin trapping molecules are rather simple in structure, but they impart rich chemical and biological properties that allow them to act as relevant pharmacological agents [15]. In ischemic conditions or malignant processes where oxygen imbalance is essentially demonstrated, spin trapping is totally involved [16]. Due to their redox properties, nitrous rivets can react with various free radicals [17,18], decomposing to NO after their addition to the released O2^•−^ [19].

Recent data have demonstrated the beneficial properties of spin trapping in the context of cardioprotection; however, the mechanism of protection against nitrons is not fully known. According to more recent reports, PBN may also inhibit expression levels of heat shock proteins, cyclooxygenase-2 (COX-2), c-Fos, and induced nitric oxide synthase (iNOS) when used in some ROS-induced disorders [20,21]. To learn what else PBN could modulate and how it may exert protective effects on myogenic cells cultured in vitro, we decided to investigate the PBN modification of medium used for SkMDS/PCs in vitro culture and characterize its biological pro-regenerative potential.

First, we assessed the effect of PBN on the basic myogenic function and phenotype of SkMDS/PCs. This was necessary for further research, requiring a relatively pure CD-56-positive SkMDS/PCs population (undifferentiated) and a sufficiently large number of cells capable of division. Very often, application of SkMDS/PCs indicated that rather high numbers of cells (≥1 × 10^8^) are required to obtain positive therapeutic effects or to ameliorate a patient’s clinical symptoms. Thus, the potential for in vitro cultured SkMDS/PC populations to achieve clinical relevance requires an optimized protocol [22]. An interesting observation from the study was the statistically significant positive effect of PBN conditioning on SkMDS/PCs phenotyping by using the CD56 marker under hypoxic conditions (*p* < 0.01) (under standard in vitro culture conditions we observed a rather neutral effect). Comparing in vitro culture under standard and hypoxic conditions without PBN we observed a decrease in the distribution of the CD 56 marker in hypoxia (an equal number of cells in analyzed samples), whereas adding an antioxidant to the culture appeared to neutralize the negative effect of hypoxia upregulating CD56-positive cells (*p* < 0.001). Thus, we have confirmed that the dose of antioxidant used did not result in any harmful effects on the human population of SkMDS/PCs cultured in vitro; on the contrary, it eliminated the negative influence of hypoxia. 

SkMDS/PCs previously conditioned with PBN, then treated with a differentiating medium including the same antioxidant, showed a greater statistically significant capacity for cell fusion, which in this case was more prominent under the hypoxic conditions of in vitro culture (*p* < 0.001). The ability to form myotubes is an important functional feature in the context of repairing the post-infarction heart in vivo. We are inclined to conclude that it is possible to produce a more functional contractile apparatus when using novel protocol for culturing SkMDS/PCs in vitro. Until now, a myocardial therapy with PBN has been tried by directly injecting antioxidant into the failing heart, which has permitted the scavenging of accumulated free radicals but has not necessarily contributed to an acceleration of heart regeneration. Treatment with the ROS scavenger α-phenyl-N-tert-butyl nitrone has significantly reduced ROS production rates at 6 weeks after transverse aortic constriction. Despite the reduction of ROS secretion, no differences in myocardial contractile functions have been observed between the examined animals [23]. Nevertheless, our proposed protocol could combine the functional and phenotypic improvement of cells for better heart in situ regeneration. During the standard characterization of the SkMDS/PC population, the most frequently troublesome determinant is the aging of the cell population. We should be able to predict the proliferation potential of human SkMDS/PCs and the level of their apoptosis, to foresee the effect of the administered cells on, for example, myocardium. The senescent process of stem/progenitor myogenic cells following their apoptosis and influencing the molecular background of organ regeneration was revealed in previous clinical trials, which showed that antioxidants may help detoxify free radicals and pro-oxidants, glutathione biosynthesis genes, control expression of redox chromatin remodeling, and ultimately inflammatory gene expression [24]. The cell aging process is correlated with mitochondrial superoxide production, and increases with the replicative age of the cell. Reduced mitochondrial superoxide generation slows down telomere shortening and delays the formation of telomeric γ-H2A.X foci. This indicates mitochondrial influence on so-called replicative senescence [25]. Therefore, we expected a positive effect of the antioxidant on the cell aging process. We demonstrated a statistically significant increase in the proportion of young cells (*p* < 0.05) in PBN-treated SkMDS/PCs under hypoxia compared to the WT non-treated cell population.

So far, several reports have dealt with the problem of the cell death and its prevention through the antioxidant addition. The protective effect of PBN on retinal cells in a rat model subjected to a degenerative factor has been previously reported [26]. In the case of in vitro studies, we are the first to demonstrate a positive influence of PBN on human myogenic cell populations. We have demonstrated a statistically significant decrease in the percentage of apoptotic cells resulting from antioxidant treatment under both standard and hypoxic in vitro culture conditions. Senescence assaying and apoptotic tests may allow us to predict positive results under in situ conditions reflecting those prevailing in the post-infarction heart.

The PBN antioxidant may be also implicated in reactions at the molecular level, and the effect of a synthetic antioxidant on certain genes and protein expression has been reported previously. For example, NF-κB translocation and COX III expression were affected when PBN protected against light-induced retinal damage in a rat model [26]. Treatment with in vitro cultured astrocytes (immediately after trauma) and addition of PBN antioxidant (100 μm) significantly lowered a trauma-induced increase in p-NKCC1 levels [27]. It has been shown that PBN addition may lead to a change in the molecular scenario via ROS elimination. Therefore, we wanted to check the effect of PBN addition to in vitro cultured cells on the gene expression of the other antioxidants (e.g., genes coding for superoxide dismutases (*SOD1*, *SOD2*, *SOD3*) and catalase). All the genes under study decreased their expression under hypoxic conditions (i.e., those imitating post-infarction myocardium). Through PBN conditioning, we were able to eliminate the negative effect of oxygen deprivation in hypoxia (in respective genes expressions studied). In respect to the *SOD1* and *SOD3* genes, we have shown that PBN addition in hypoxia triggered a higher expression of these genes than in the WT control population.

Nuclear translocation of the forkhead box O transcription factor (FoxO) (homolog DAF-16) through differences in the mechanisms of its activation caused by antioxidants and oxidants action extended the lifespan of *Caenorhabditis elegans*. It was discovered that the combination of early antioxidant and late anti-oxidation therapy was the most effective way to lengthen a lifespan of *C. elegans* [28]. Based on these data, we decided to assess the effect of PBN on anti-aging genes in human SkMDS/PCs. We studied the participation of *FOXO* gene expression and *SIRT* gene expression in the regulation of cell-aging and mammalian longevity. As an example, the brain-specific Sirt1-overexpresed (BRASTO) in transgenic mice, showing significant lifespan extension in both sexes, while aged BRASTO mice exhibited phenotypes consistent with those observed in aging delay [29]. Our study demonstrated a positive influence of hypoxia on *FOXO* and *SIRT* overexpression (*p* < 0.01) compared to WT SkMDS/PCs cultured under standard in vitro conditions; however, PBN conditioning did not provide a clear beneficial effect on the cell populations under study.

In our next approach we assessed expression of the anti-apoptotic gene *BCL2* and the *Hsp70* gene, which are suspected to regulate a cellular stress. PBN conditioning significantly increased *BCL2* gene expression under standard in vitro culture conditions, and decreased *Hsp70* gene expression under both standard and hypoxic conditions of in vitro culture. It could be therefore cautiously concluded that PBN exerted some protective effect in respect to the well-being of human SkMDS/PCs. A protective effect of antioxidants on spermatogonial stem cells was also observed in another study in which exogenous antioxidant (α-tocopherol) was added to the cryoprotective medium, leading to a significantly (*p* < 0.05) lower expression of pro-apoptotic gene *Bax* and a significant rise in *Bcl2* expression in cells after thawing [30]; this may confirm the results obtained in respect to *Bcl2* gene expression. On the other hand, the *Hsp70* coding gene has been considered a biomarker of oxidative injury [31,32], which assures us that its decreased expression after adding to the culture of PBN antioxidant was beneficial to the cells.

Antioxidant enzymes and ROSs play significant roles during the differentiation of spermatogonial stem cells (SSCs) and SkMDS/PCs. In order to effectively regenerate damaged muscle tissue, the inflammatory reaction must be strictly followed by the restoration of cellular architecture [33]. At each stage of muscle myogenesis, muscle satellite cells and myoblast functions are strictly regulated by the expression of muscle regulatory transcription factors, activated in a specific order (MRFs-basic helix-loop-helix transcription factors: Myf5, MYOD, MYOG, Myf6) [34]. We demonstrated the effect of antioxidant treatment on early (*MYOD*) and late (*MYOG*) myogenic gene expression, and observed a statistically significant increase in both myogenic gene expressions in SkMDS/PCs conditioned with PBN medium under hypoxic conditions vs. the non-treated cell populations. Nonetheless, interpretation of these results can be ambiguous. Indeed, overexpression of early transcription factor (*MYOD*) is likely related to SkMDS/PC function and proliferation. This therefore points to a positive aspect of such phenomenon in young, non-differentiated cell populations that are flexible and able to proliferate, whereas high expression of *MYOG* at the same time might be at least neutral, or may participate in cell differentiation (to myotubes), which is essentially associated with the formation of muscle fibers. Whether these factors cooperate should next be investigated on the protein level; however, this has undoubtedly been positively associated with our functional study herein performed.

The last aspect we would like to outline is the impact of PBN on the cell aging, assessed at the DNA level. We examined the length of telomeres using the real-time PCR method. Current scientific reports point out to the effect of physical stress (physical activity) on the length of telomeres [35], which may involve exposure or elimination of free radicals. The lack of research on an antioxidant’s influence on the length of telomeres in human SkMDS/PCs prompted us to evaluate this aspect. We discovered that statistically significant longer telomeres structures in SkMDS/PCs cultured in vitro with PBN under hypoxic conditions compared to the other cell populations under study (*p* < 0.05). This could suggest a renewal of the cell proliferation capacity and function.

The positive effect of PBN on gene expression prompted us to test the expression respective protein products. So far, studies in a mouse model have shown the effect of PBN on the expression of single molecules such as Sgk1 in brain tissue [36], and RPE65 activity in the retina [37]. We decided to test this comprehensively, primarily with respect to antioxidant factors such as CAT, SOD1 or THX. As often observed earlier in our studies, expression at the gene level does not often predict protein expression. In this case, the expression trend was maintained. However, we showed some results with statistical significance. We observed a greater amount of catalase, SOD1 and TRX in cell populations preconditioned with PBN and cultured under hypoxic conditions compared to wild-type cells cultured under standard in vitro conditions (*p* < 0.05) (Figure 8). PBN by itself significantly increased the amount of TRX protein under standard culture conditions (*p* < 0.05). The amount of HSP70 protein, however, increased during PBN preconditioning under standard in vitro culture conditions (*p* < 0.001) and hypoxia (*p* < 0.01) (Figure 8).

## 5. Conclusions

In this study, the biological characterization and molecular analysis of human SkMDS/PCs were performed, which indicated that PBN conditioning has potential to improve in vitro stem/progenitor cell preparation protocol for prospective cellular therapy. Cells conditioned by PBN seemed to possess better proliferation potential, with high levels of young cells, low percentages of apoptosis, and increased telomere lengths. At the same time, myogenic cells did not lose their phenotypes and maintained other antioxidant gene expressions, underlining their molecular predisposition for anti-apoptotic phenotypes. PBN-treated human SkMDS/PCs adapted well to hypoxic conditions, and could be easily propagated for post-infarction heart cellular interventions.

## Figures and Tables

**Table 1:** Primer sequences and the expected lengths of synthesis products.

	Sequence (5′->3′)	Length of the PCR Product
*CAT*		
Forward primer	TATCCTGACACTCACCGCCA	277
Reverse primer	CGTTCACATAGAATGCCCGC	
*BCL2*		
Forward primer	GGATAACGGAGGCTGGGATG	123
Reverse primer	TATTTGTTTGGGGCAGGCAT	
*FOXO1*		
Forward primer	GAGGGTTAGTGAGCAGGTTAC	243
Reverse primer	TGGCACAGTCCTTATCTACAG	
*Hsp70*		
Forward primer	TTGACTGTGTTGTTTCGGTTCC	290
Reverse primer	TCTACCTCCCAATGTCGTGT	
*SIRT1*		
Forward primer	TGGTTTGCGTCGTAGTCTCC	168
Reverse primer	GTCCATTACTTTCCTTCTGCTC	
*SOD2*		
Forward primer	ACCTGCCCTACGACTACGG	262
Reverse primer	AACTCCCCTTTGGGTTCTCC	
*SOD3*		
Forward primer	ATGCTGGCGCTACTGTGTTC	100
Reverse primer	ACTCCGCCGAGTCAGAGTT	
*MYOD*		
Forward primer	ACGGCATGATGGACTACAG	212
Reverse primer	CGACTCAGAAGGCACGTC	
*MYOG*		
Forward primer	GCTGTATGAGACATCCCCCTA	226
Reverse primer	CGACTTCCTCTTACACACCTT	

**Table 2:** Primers’ sequences and expected length of synthesis product.

	Sequence (5′->3′)	Length of the PCR Product
*ALB*		
Forward primer	TTTGCAGATGTCAGTGAAAGAGA	61
Reverse primer	TTTGCAGATGTCAGTGAAAGAGA	
*Telo*		
Forward primer	ACACTAAGGTTTGGGTTTGGGTTTGGGTTTGGGTTAGTGT	61
Reverse primer	GTTAGGTATCCCTATCCCTATCCCTATCCCTATCCCTAACA	

## Data Availability

All data presented in this study are openly available in this paper.
